# Evaluating Policy Changes for Adjusting Payment to Address Health Disparities

**DOI:** 10.1001/jamahealthforum.2024.2905

**Published:** 2024-09-13

**Authors:** W. Ryan Powell, Luke Chamberlain, William R Buckingham, Ying Cao, Mariétou H. Ouayogodé, Robin L. Lankton, Amy J. H. Kind

**Affiliations:** 1 University of Wisconsin Center for Health Disparities Research (CHDR), Madison; 2 Division of Geriatrics and Gerontology, Department of Medicine, University of Wisconsin School of Medicine and Public Health, Madison; 3 Department of Population Health Sciences, University of Wisconsin School of Medicine and Public Health, Madison; 4 Office of Population Health, UW Health, Madison, Wisconsin

## Abstract

This study analyzes area-level Health Equity Benchmark Adjustment formula changes to demonstrate how policy choices result in resource reallocation.

## Introduction

New federal initiatives signal a growing intent to align payment and policies to address social needs.^1^ The Center for Medicare and Medicaid Innovation (CMMI) should be commended for pioneering new models that address disparities, but it is just as important that changes direct resources to patients with highest need.

As example, CMMI’s Accountable Care Organization Realizing Equity, Access, and Community Health (ACO REACH) model includes a Health Equity Benchmark Adjustment (HEBA) using individual- and area-level social formula components.^2,3^ The HEBA provides additional money to ACOs caring for beneficiaries with the highest HEBA rankings and a reduction with the lowest HEBA rankings. In program year (PY) 2023, the HEBA formula used a 100% national Area Deprivation Index^4^ as its area-level metric.^2^ “In response to stakeholder feedback” and “to better identify underserved beneficiaries living in high cost-of-living areas” CMMI changed to a blended 50% national/50% state Area Deprivation Index metric in PY2024.^3^ To demonstrate how policy choices result in resource reallocation, we analyzed area-level HEBA formula changes.

## Methods

Census block groups ranking higher (denoting higher neighborhood-level socioeconomic disadvantage) in PY2024 compared to PY2023 are likely to experience financial gains (conversely losses are more likely for lower rankings). Importantly, this reflects a mathematical reranking rather than an actual change to underlying social circumstances in each block group.

HEBA area-level financial (2024 deciles in eTable 1 in Supplement 1) and rank changes for all census block groups were analyzed. Rank changes within the 50 largest metropolitan areas were also analyzed. Data sources and sample derivation are detailed in eTables 2 and 3, respectively, in Supplement 1. This study did not require institutional review board approval, given the use of publicly available data only, and followed STROBE reporting guidelines.

## Results

### Financial Gains and Losses

**Figure 1.**
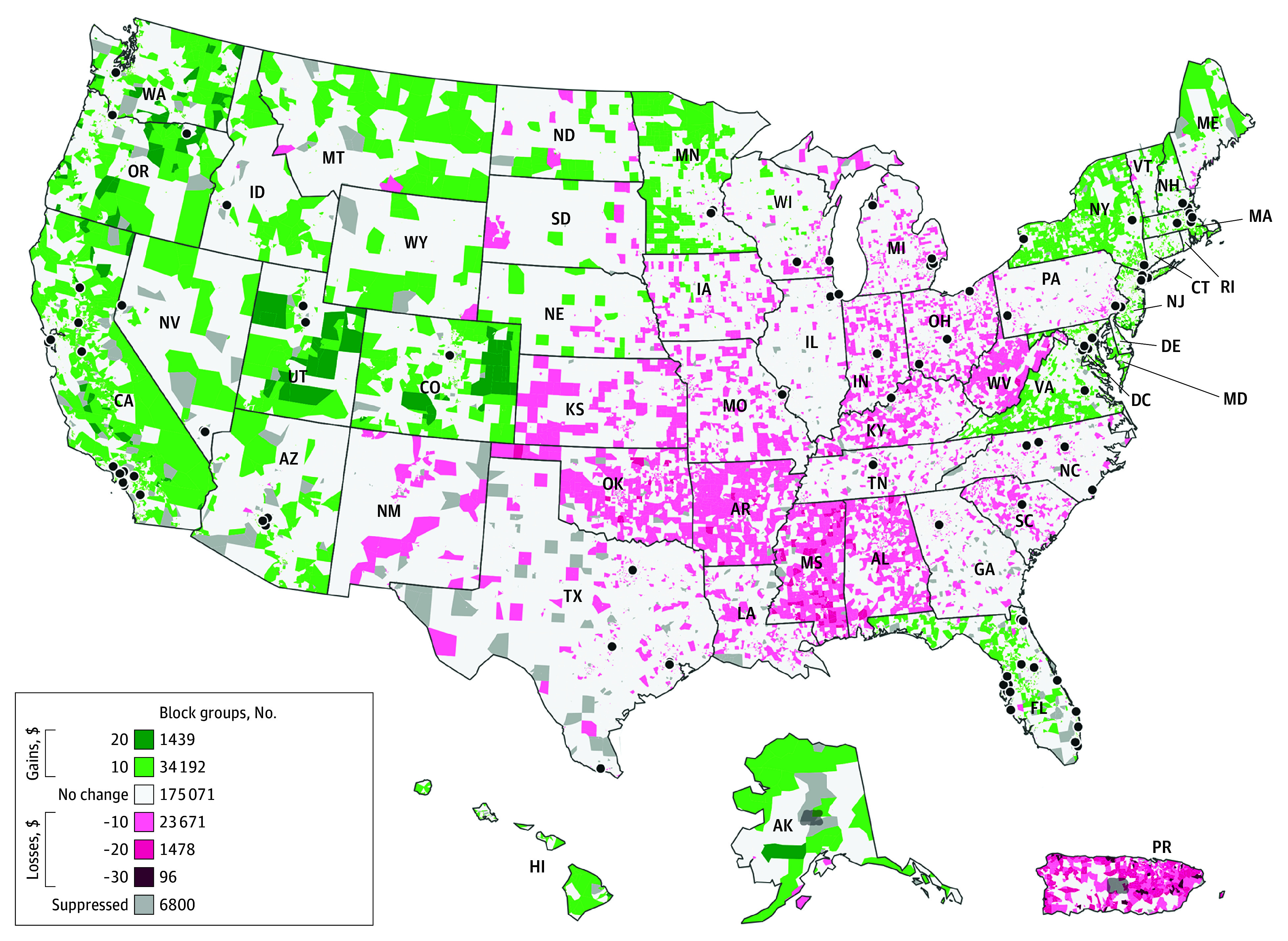
Financial Gains and Losses Across the Accountable Care Organization Realizing Equity, Access, and Community Health (ACO REACH) Model’s Health Equity Benchmark Adjustment (HEBA) Area-Level Rank Changes, 2023-2024 Areas in green represent census block groups where the program year (PY) 2024 HEBA formula (blended national/state Area Deprivation Index score) resulted in more per-beneficiary-per-month HEBA dollars than in the PY2023 formula (national Area Deprivation Index only) for ACO REACH programs serving beneficiaries residing in those census block groups. Likewise, areas in pink represent census block groups where the PY2024 HEBA formula resulted in a fewer dollars per beneficiary per month for ACO REACH programs serving beneficiaries residing in those census block groups. Gray areas are suppressed due to block groups containing any of the following: fewer than 100 people, fewer than 30 housing units or more than 33% of the population living in group quarters, and census data labeled as not available or missing in the core component variables. Map dots represent 2023 ACO REACH model sites.

Of 235 947 block groups, 25.8% had a financial adjustment upward or downward based on policy changes (Figure 1). Areas with the highest percentages of block groups facing negative financial adjustments were Puerto Rico (1824 of 2466 [74.0%]), Mississippi (1433 of 2389 [60.0%]), West Virginia (868 of 1605 [54.1%]), Arkansas (1202 of 2248 [53.5%]), and Oklahoma (1357 of 3222 [42.1%]). Areas with the highest percentages of block groups with positive financial adjustments were California (11 350 of 25 215 [45.0%]), Hawaii (441 of 1011 [43.6%]), Washington, DC (209 of 534 [39.1%]), Massachusetts (1894 of 4953 [38.2%]), and New York (5507 of 15 257 [36.1%]).

### Rank Gains and Losses Across the 50 Largest Metropolitan Areas

**Figure 2.**
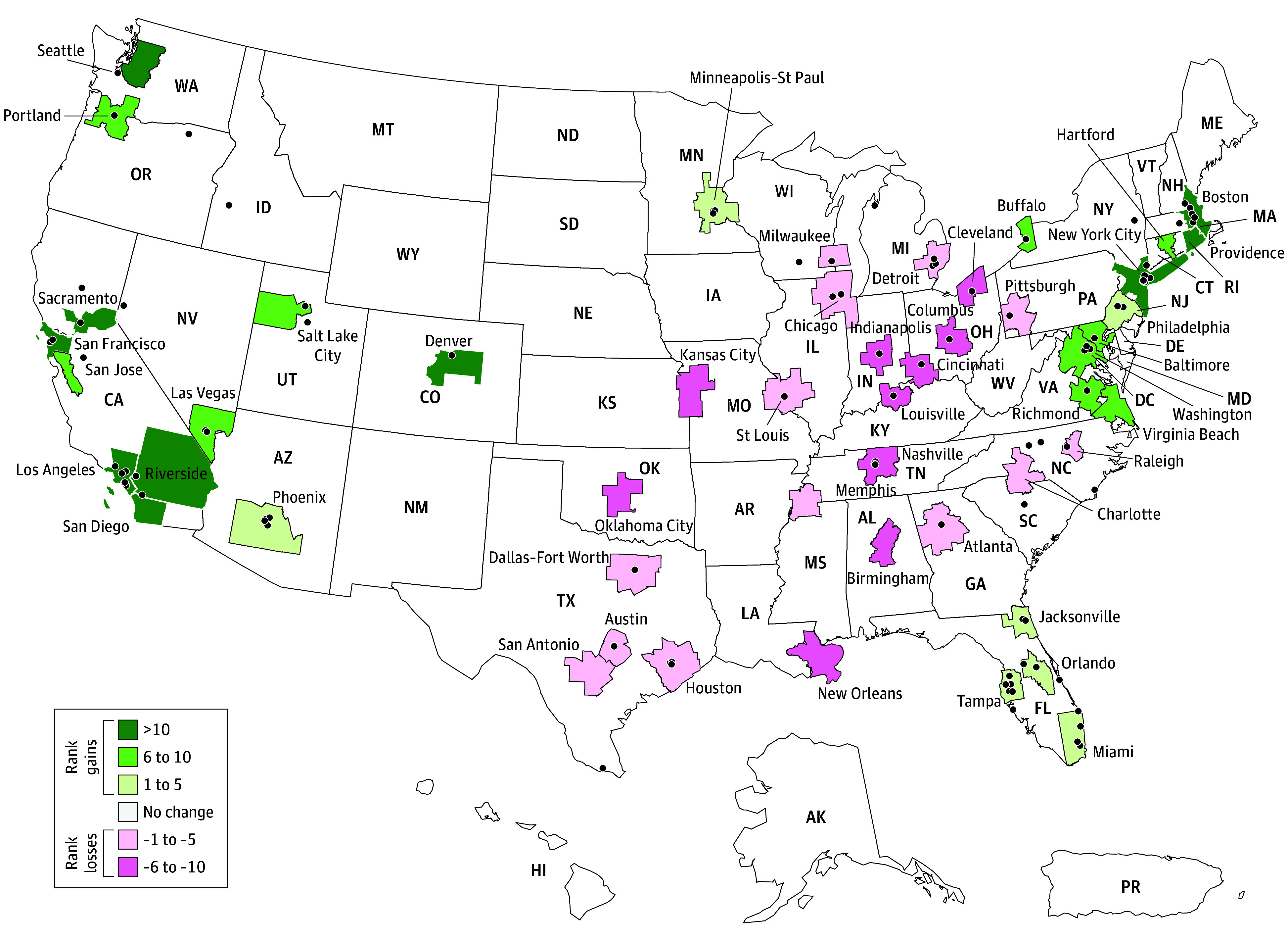
Average Accountable Care Organization Realizing Equity, Access, and Community Health (ACO REACH) Model Health Equity Benchmark Adjustment (HEBA) Area-Level Rank Changes Within the 50 Largest Metropolitan Areas, 2023-2024 Green metropolitan areas are areas where the program year (PY) 2024 formula (blended 50% national/50% state Area Deprivation Index) resulted in a higher average HEBA rank compared to the PY2023 HEBA (which used national Area Deprivation Index alone) and where additional resources were more likely to be assigned. Pink metropolitan areas represent areas where the PY2024 HEBA formula resulted in a lower average HEBA rank compared to the PY2023 formula (less likely to be assigned additional resources).

Overall, 23 of 50 (46%) metropolitan areas experienced rank losses on average (Figure 2). New York and Los Angeles combined had 99.7% (22 753 of 22 811) of their block groups ranked higher in the PY2024 formula. The next 3 largest metropolitan areas demonstrated the opposite, having the majority of their block groups rank lower: Chicago (4924 of 6803 [72.4%]), Dallas (3742 of 4260 [87.8%]), and Houston (3484 of 4099 [85.0%]).

## Discussion

The 2024 ACO REACH HEBA changes generally benefit Northeastern and Western coastal states and cities, while the South and Midwest fare less well. Measured against the Federal Medical Assistance Percentage^5^ as an accepted standard of state average per-capita income, these shifts are in the opposite direction of need nationally. In addition, the PY2024 area-level HEBA change helps and hurts nearly the same number of large metropolitan areas. It is possible that HEBA individual factors could mitigate some of the resource shifts seen in this analysis, but approximately 85% of Medicare ACO beneficiaries are neither dually eligible nor beneficiaries of low-income subsidy^6^; therefore, their entire HEBA is based on area-level components.

On July 26, 2024, CMMI announced that it will replace the PY2024 blended national/state ADI with a new area-level ACO REACH measure in PY2025. As CMMI continues to test new equity adjustments, it is important that changes be grounded in scientific principles with extensive testing and validation to ensure the tightest linkage to social needs and health outcomes for underserved communities across the entire US. Stakeholder input is important, but not all stakeholders have the same policy influence. Decisions based on smaller geographies or specific metropolitan areas in isolation of the entire nation should be avoided. A robust scientific framework could broadly inform policy across CMMI programs.

## Notes

Supplement 1. **eTable 1.** ACO REACH 2023 and 2024 Health Equity Benchmark Adjustments **eTable 2.** Data Sources **eTable 3.** Sample Derivation

Supplement 2. Data Sharing Statement
