# Supplementary material for: Evaluating Policy Changes for Adjusting Payment to Address Health Disparities
Source: JAMA Health Forum. 2024 Sep 13;5(9):e242905. doi: 10.1001/jamahealthforum.2024.2905 (PMC11400218; doi:10.1001/jamahealthforum.2024.2905)
Supplement: Supplement 1. — eTable 1. ACO REACH 2023 and 2024 Health Equity Benchmark Adjustments eTable 2. Data Sources eTable 3. Sample Derivation [file jamahealthforum-e242905-s001.pdf]

Supplemental Online Content

Powell WR, Chamberlain L, Buckingham WR et al. Evaluating policy changes for adjusting payment to address health disparities. *JAMA Health Forum*. 2024;5(9):e242905. doi:10.1001/jamahealthforum.2024.2905

eTable 1. ACO REACH 2023 and 2024 Health Equity Benchmark Adjustments
eTable 2. Data Sources
eTable 3. Sample Derivation

This supplemental material has been provided by the authors to give readers additional information about their work.

© 2024 Powell WR et al. *JAMA Health Forum.*

eTable 1. ACO REACH 2023 and 2024 Health Equity Benchmark Adjustments

Components of Composite Measure	PY 2023	PY 2024
National ADI	80%	33%
State ADI	0%	33%
Dual Eligible and Low Income Subsidy*	20%	33%

*Note: In Low Income Subsidy added in PY 24. PY 23 used Dual Eligibility alone.

PY: Performance Year; ADI: Area Deprivation Index.

Beneficiary Decile	1	2	3	4	5	6	7	8	9	10
Dollar ($) adjustment per declile, PY 2023	-6	-6	-6	-6	-6	0	0	0	0	30
Dollar ($) adjustment per declile, PY 2024	-10	-10	-10	0	0	0	0	10	20	30

The Area Deprivation Index (ADI) is a neighborhood-level (census block group level) composite index reflecting 17 factors in the American Community Survey on income, education, employment and housing via principal components methodology. The resulting score is transformed into a percentile ranking based on the comparator group: either comparing block groups to the entire nation (national ADI) or within an individual state, including the District of Columbia and Puerto Rico (state ADI). For both state and national measures, high rankings denote living in one of the most socioeconomic disadvantaged neighborhoods.

The CMMI changed their PY24 HEBA to equally weight the national ADI ranking with the state ADI ranking and added low-income subsidy to dual eligibility. The ADI components make up the majority of a beneficiary's total score, from 100% (for non-Medicaid beneficiaries) to 80% (for Medicaid beneficiaries) in PY23, and 100% (for non-Medicaid, non-Low Income Subsidy beneficiaries) to 66% (for Medicaid and/or Low Income Subsidy beneficiaries) in PY24. The CMMI also changed the positive and negative per-beneficiary-per-month (PBPM) amounts in some decile rankings.

© 2024 Powell WR et al. *JAMA Health Forum.*

eTable 2. Data Sources

Source Data	Year	Notes	Reference
Shapefile for Mapping	2021	Simplified linework using https://mapshaper.org/	Manson S, Schroeder J, Van Riper D, Knowles K, Kugler T, Roberts F, Ruggles S. IPUMS National Historic Geographic Informations System version 18.0 [US Block Groups 2021]. Minneapolis, MN: IPUMS. 2023. Available from: http://doi.org/10.18128/D050.V18.0
Metropolitan Areas (CBSA)	2021	Core-Based Statistical Area (CBSA) definition	U.S. Census Bureau. 2021 TIGER/Line Shapefiles; 2021. Available from: https://www2.census.gov/geo/tiger/TIGER2021/CBSA/
Population Density		Based on column in the CBSA Shapefile: "Pop Estimate 2021"	
US Census Block Groups	2021	Centroid within polygon used to classify inside or outside CBSAs	U.S. Census Bureau. 2021 TIGER/Line Shapefiles; 2021. Available from: https://www.census.gov/geographies/mapping-files/time-series/geo/tiger-line-file.2021.html#list-tab-790442341
Area Deprivation Index	2021	National and State Rankings	-Kind AJH, Buckingham W. Making Neighborhood Disadvantage Metrics Accessible: The Neighborhood Atlas. N Engl J M, 2018. 378: 2456-2458. DOI: 10.1056/NEJMp1802313. PMCID: PMC6051533.' -University of Wisconsin School of Medicine and Public Health. 2021 Area Deprivation Index version 4 [Data file]: Madison(WI); 2021. Available from: https://www.neighborhoodatlas.medicine.wisc.edu/

© 2024 Powell WR et al. *JAMA Health Forum.*

eTable 3. Sample Derivation

	Included N	Included %	Excluded N	Excluded %
**Total Block Groups in US**	242,747	*100.0%*		
**Exclusions:**			**6,800**	*2.8%*
**NS: Not in 50 States, District of Columbia, or Puerto Rico**[a]			412	*0.2%*
**Block Group Suppressed**			6,388	*2.6%*
**PH: Low Population/Housing**			2,639	*1.1%*
**GQ: High Group Quarters**			2,729	*1.1%*
**Both PH-GQ**			768	*0.3%*
**Questionable Data Integrity**[b]			252	*0.1%*
**Block Group Denominator Used for Analysis**	**235,947**	*97.2%*		
**Metropolitan Areas #1-50**	120,932	*51.3%*		
**New York City & Los Angeles**[c]	22,811	*9.7%*		
**Metro Areas #3-50**	98,121	*41.6%*		
**Outside Metropolitan Areas #1-50**	115,015	*48.7%*		

[a] Exclusion criteria includes block groups that are fully in lakes, rivers, or the ocean, and US territories other than Puerto Rico

[b] Block groups without an ADI due to Questionable Data Integrity, stemming from missing data in the source ACS data.

[c] According to Core Based Statistical Areas (CBSAs): 35620 and 31080

Note: The centroid of the block group was used to assign block groups to inside and outside metropolitan areas

© 2024 Powell WR et al. *JAMA Health Forum.*
