# Supplementary material for: Evaluating Policy Changes for Adjusting Payment to Address Health Disparities
Source: JAMA Health Forum. 2024 Sep 13;5(9):e242905. doi: 10.1001/jamahealthforum.2024.2905 (PMC11400218; doi:10.1001/jamahealthforum.2024.2905)
Supplement: Supplement 2. — Data Sharing Statement [file jamahealthforum-e242905-s002.pdf]

Data Sharing Statement

Powell. Evaluating Policy Changes for Adjusting Payment to Address Health Disparities. *JAMA Health Forum*. Published September 13, 2024. doi:10.1001/jamahealthforum.2024.2905

Data
Data available: No
